# Impact of decreased levels of total CO2 on in-hospital mortality in patients with COVID-19

**DOI:** 10.1038/s41598-023-41988-4

**Published:** 2023-10-04

**Authors:** Yaerim Kim, Soie Kwon, Seong Geun Kim, Jeonghwan Lee, Chung-hee Han, Sungbong Yu, Byunggun Kim, Jin Hyuk Paek, Woo Yeong Park, Kyubok Jin, Seungyeup Han, Dong Ki Kim, Chun Soo Lim, Yon Su Kim, Jung Pyo Lee

**Affiliations:** 1 Department of Internal Medicine, Keimyung University School of Medicine, Daegu, Korea; 2 Department of Internal Medicine, Chung-Ang University Heukseok Hospital, Seoul, Korea; 3 Department of Internal Medicine, Inje University Sanggye Paik Hospital, Seoul, Korea; 4 Department of Internal Medicine, Seoul National University Boramae Medical Center, Seoul, Korea; 5 Department of Obstetrics and Gynecology, Bagae Hospital, Pyeongtaek, Gyeonggi-Do Korea; 6 Department of General Surgery, Bagae Hospital, Pyeongtaek, Gyeonggi-Do Korea; 7 Department of Orthopedic Surgery, Bagae Hospital, Pyeongtaek, Gyeonggi-Do Korea; 8 Department of Internal Medicine, Seoul National University Hospital, Seoul, Korea; 9 Department of Internal Medicine, Seoul National University College of Medicine, Seoul, Korea

**Keywords:** Medical research, Nephrology

## Abstract

Decreased total CO_2_ (tCO_2_) is significantly associated with all-cause mortality in critically ill patients. Because of a lack of data to evaluate the impact of tCO_2_ in patients with COVID-19, we assessed the impact of tCO_2_ on all-cause mortality in this study. We retrospectively reviewed the data of hospitalized patients with COVID-19 in two Korean referral hospitals between February 2020 and September 2021. The primary outcome was in-hospital mortality. We assessed the impact of tCO_2_ as a continuous variable on mortality using the Cox-proportional hazard model. In addition, we evaluated the relative factors associated with tCO_2_ ≤ 22 mmol/L using logistic regression analysis. In 4,423 patients included, the mean tCO_2_ was 24.8 ± 3.0 mmol/L, and 17.9% of patients with tCO_2_ ≤ 22 mmol/L. An increase in mmol/L of tCO_2_ decreased the risk of all-cause mortality by 4.8% after adjustment for age, sex, comorbidities, and laboratory values. Based on 22 mmol/L of tCO_2_, the risk of mortality was 1.7 times higher than that in patients with lower tCO_2_. This result was maintained in the analysis using a cutoff value of tCO_2_ 24 mmol/L. Higher white blood cell count; lower hemoglobin, serum calcium, and eGFR; and higher uric acid, and aspartate aminotransferase were significantly associated with a tCO_2_ value ≤ 22 mmol/L. Decreased tCO_2_ significantly increased the risk of all-cause mortality in patients with COVID-19. Monitoring of tCO_2_ could be a good indicator to predict prognosis and it needs to be appropriately managed in patients with specific conditions.

## Introduction

Low levels of serum bicarbonate usually indicate metabolic acidosis or renal compensation for respiratory alkalosis. Metabolic acidosis is relatively common among seriously ill patients, especially in subjects with major organ dysfunction, and is closely associated with increased mortality^1–4^. The presence of metabolic acidosis affects diverse organ systems, and the cardiovascular system is the organ most critically affected, exhibiting a change in vascular resistance and cardiac output depending on the level of pH^5^. Moreover, metabolic acidosis leads to increased inflammation and interleukin stimulation via macrophage production and impaired immune response^6^. In addition to metabolic acidosis, respiratory distress reduces the level of serum bicarbonate. This could reflect various responses that are complexly intertwined depending on patients' underlying conditions and problems. Therefore, it might be necessary to intervene based on a detailed assessment, especially for critically ill patients.

To obtain information on acid–base status, including serum bicarbonate levels, arterial blood gas analysis is primarily used. However, a relatively invasive technique is required to obtain blood, and detailed processing is essential^7^. Instead of serum bicarbonate, measuring serum total carbon dioxide (tCO_2_) could be a good method to assess acid–base status, and serum tCO_2_ exhibits a significant correlation with bicarbonate concentration^8^. Serum tCO_2_ also has the strength of a convenient measurement approach, which can be done along with the measurement of serum creatinine and electrolytes using a biochemical analyzer. In this regard, serum tCO_2_ could be a useful option for the assessment of a specific group with infectious diseases showing high transmission infectivity, such as that associated with novel coronavirus disease (COVID-19) infection.

After the outbreak of COVID-19 in December 2019, approximately 6 million people died worldwide^9^. Although mortality differed across countries and pandemic periods, the case-fatality rate was reported to range from 0.4 to 15%^10^. The mortality rate was higher among hospitalized patients with older age, decreased oxygen saturation, elevated levels of C-reactive protein (CRP), and underlying disease^11,12^. Acidosis also has a role in dysregulation of the immune system and multidirectional inflammatory reactions, and it is regarded as a factor related to the pathogenesis of severe forms of COVID-19 infection^13^. In addition, COVID-19 infection is closely associated with respiratory failure characterized by desaturation, hypercapnia, and acid–base imbalance, as well as a poor prognosis^14^. However, there was a lack of data to show the exact association between metabolic acidosis status and mortality risk in patients with COVID-19.

As a significant risk factor and confounding factor, we aimed to evaluate the impact of decreased levels of tCO_2_ on mortality in hospitalized patients with COVID-19 in this study. Moreover, we tried to identify the factors related to decreased tCO_2_ based on the cutoff value representing an increased mortality risk in this population.

## Results

### Baseline characteristics

**Figure 1:**
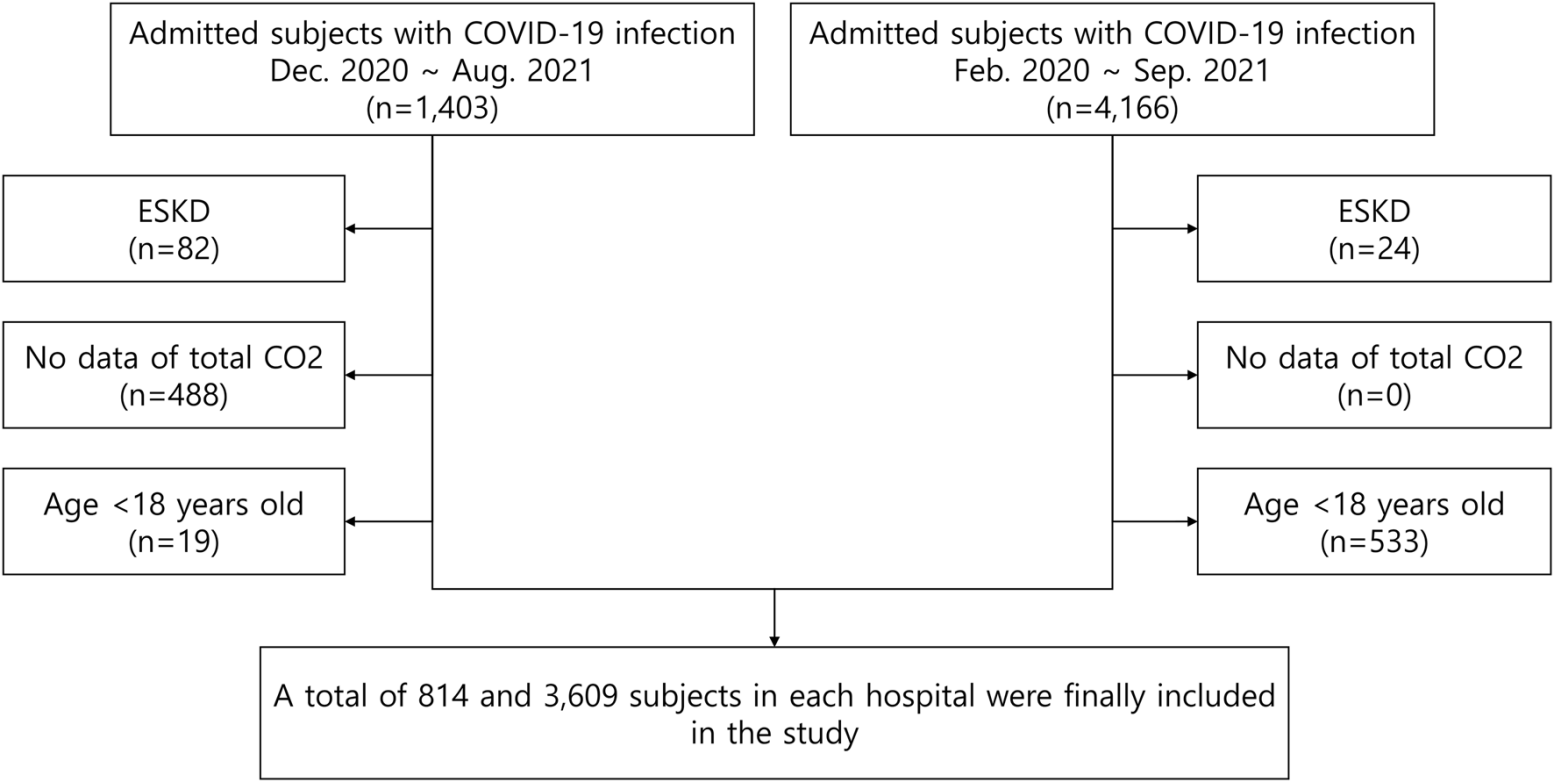
Flow diagram for the study populations. ESKD, end-stage kidney disease; CO2, carbon dioxide.

A total of 4,423 patients were included in this study (Fig. 1). There were 792 (17.9%) patients with tCO_2_ ≤ 22 mmol/L at the time of admission to the hospital due to COVID-19 infection. In the comparison of the two groups according to the cutoff of 22 mmol/L tCO_2_, patients with lower tCO_2_ showed older age; a higher prevalence of males; higher white blood cell (WBC) count, aspartate aminotransferase (AST), alanine aminotransferase (ALT), uric acid, and CRP; and lower hemoglobin, platelet, serum calcium, serum albumin, and lower potassium. The prevalence of comorbidities such as hypertension, diabetes, cerebrovascular disease, and cardiovascular disease was higher in patients with lower tCO_2_ (Table 1). There were 17 patients with acute kidney injury requiring kidney replacement therapy (KRT), and it was more frequent in patients with lower tCO_2_.

**Table 1:** Baseline characteristics according to the tCO_2_ level.

Variables	Total (n = 4423)	tCO_2_ > 22 (n = 3631)	tCO_2_ ≤ 22 (n = 792)	*P* value
Age, year	54.7 ± 18.3	53.8 ± 18.0	58.7 ± 19.1	< 0.001
Sex, male, n (%)	1,935 (45.7)	1,527 (44.4)	408 (51.5)	< 0.001
WBC, × 1,000/uL	5.3 ± 2.5	5.1 ± 2.3	5.8 ± 3.1	< 0.001
Hemoglobin, mg/dL	13.4 ± 1.8	13.5 ± 1.7	13.0 ± 2.0	< 0.001
Platelet, × 1,000/uL	193.9 ± 73.5	195.8 ± 73.1	185.3 ± 74.8	< 0.001
Calcium, mg/dL	8.7 ± 0.9	8.7 ± 0.8	8.4 ± 1.0	< 0.001
Phosphorus, mg/dL	3.2 ± 0.7	3.2 ± 0.6	3.2 ± 0.9	0.963
Potassium, mmol/L	4.0 ± 0.5	4.0 ± 0.5	4.1 ± 0.6	0.029
Glucose, mg/dL	129.2 ± 52.6	127.2 ± 50.0	138.4 ± 63.1	< 0.001
BUN, mg/dL	14.5 ± 10.7	13.5 ± 8.6	19.0 ± 16.4	< 0.001
Creatinine, mg/dL	1.0 ± 1.8	0.9 ± 1.7	1.3 ± 1.9	< 0.001
eGFR, mL/min/1.73 m2	94.2 ± 25.0	96.4 ± 22.4	84.0 ± 32.7	< 0.001
Total bilirubin, mg/dL	0.5 ± 0.4	0.6 ± 0.3	0.6 ± 0.5	0.108
AST, mg/dL	37.0 ± 33.8	35.5 ± 29.0	43.7 ± 50.0	< 0.001
ALT, mg/dL	30.7 ± 33.4	30.3 ± 33.4	32.4 ± 33.7	0.106
Protein, g/dL	6.8 ± 0.6	6.8 ± 0.6	6.7 ± 0.6	< 0.001
Albumin, g/dL	4.0 ± 0.5	4.1 ± 0.5	3.9 ± 0.5	< 0.001
Uric acid, mg/dL	2.5 ± 2.5	2.2 ± 2.5	4.0 ± 2.1	< 0.001
Total cholesterol, mg/dL	152.1 ± 38.2	153.7 ± 37.7	144.0 ± 40.0	< 0.001
C-reactive protein, mg/dL	2.9 ± 4.4	2.8 ± 4.3	3.3 ± 4.7	0.008
tCO2, mmol/L	24.8 ± 3.0	23.4 ± 4.8	20.3 ± 1.9	< 0.001
Hypertension, n (%)	522 (11.8)	385 (11.2)	110 (13.9)	< 0.001
Diabetes, n (%)	333 (7.5)	226 (6.6)	89 (11.2)	0.033
Cerebrovascular disease, n (%)	65 (1.5)	46 (1.3)	19 (2.4)	0.016
Cardiovascular disease, n (%)	51 (1.2)	36 (1.0)	15 (1.9)	0.031
Obstructive pulmonary disease, n (%)	69 (1.6)	58 (1.6)	11 (1.4)	0.668
Acute kidney injury requiring KRT, n (%)	17 (0.4)	8 (0.2)	9 (1.1)	< 0.001
In-hospital staying periods, days	11.8 ± 8.5	11.7 ± 8.2	12.4 ± 9.7	0.030

WBC, white blood cell; BUN, blood urea nitrogen; eGFR, estimated glomerular filtration rate; AST, aspartate aminotransferase; ALT, alanine aminotransferase; tCO2, total carbon dioxide; KRT, kidney replacement therapy.

We evaluated tCO_2_ level according to the underlying comorbidities including hypertension, diabetes, cerebrovascular disease, cardiovascular disease, and obstructive pulmonary disease. The level of mean tCO_2_ was significantly lower in patients with diabetes, cerebrovascular disease, and cardiovascular disease (Table S1). On the contrary, there was no difference based on hypertension or obstructive pulmonary disease.

### Impact of tCO_2_ on all-cause mortality

During the in-hospital staying periods of 11.8 ± 8.6 days, there were 92 (2.5%) and 53 (6.7%) mortality cases in the subgroup of tCO_2_ > 22 mmol/L and ≤ 22 mmol/L, respectively. Mortality cases showed older age; higher levels of inflammatory markers, such as WBC and CRP; lower levels of nutritional markers, such as serum albumin and total cholesterol; and a higher prevalence of comorbidities, including hypertension, diabetes, cerebrovascular disease, and cardiovascular disease (Table S2). Patients with acute kidney injury requiring KRT also showed higher mortality. The mean level of tCO_2_ was significantly different between the survival (24.9 ± 3.0) and mortality (23.4 ± 4.8) groups. An increase in tCO_2_ of 1 mmol/L significantly reduced the risk of death by approximately 5% (adjusted hazard ratio [aHR] 0.94, 95% confidence interval [CI] 0.90–0.98) after adjustment for laboratory results and comorbidities (Table 2). Considering the impact of lactate level on acidosis status and mortality, we performed a Cox-proportional analysis including the lactate variable. Although, there were only 18.4% of patients with lactate levels, lower tCO2 significantly increased the risk of in-hospital mortality (aHR 0.94, *P* = 0.024).

**Table 2:** Significance of tCO_2_ in terms of all-cause mortality.

	Model 1		Model 2		Model 3		Model 4	
	HR (95% CI)	*P*-value	aHR (95% CI)	*P*-value	aHR (95% CI)	*P* value	aHR (95% CI)	*P* value
tCO_2_	0.90 (0.86, 0.94)	< 0.001	0.93 (0.89, 0.97)	0.001	0.93 (0.89, 0.97)	< 0.001	0.94 (0.90, 0.98)	0.006

Model 1: Unadjusted.

Model 2: Adjusted for age and sex.

Model 3: Adjusted for variables in model 2 with comorbidities including hypertension, diabetes, cerebrovascular disease, cardiovascular disease, pulmonary disease, and acute kidney injury requiring kidney replacement therapy.

Model 4: Adjusted for variables in model 3 with laboratory variables including white blood cell count, hemoglobin, platelet, potassium, calcium, glucose, blood urea nitrogen, eGFR, protein, albumin, uric acid, total cholesterol, total bilirubin, C-reactive protein.

HR, hazard ratio; CI, confidence interval; aHR, adjusted hazard ratio; tCO2, total carbon dioxide.

### A cut-off value of tCO_2_ representing the increased mortality

**Figure 2:**
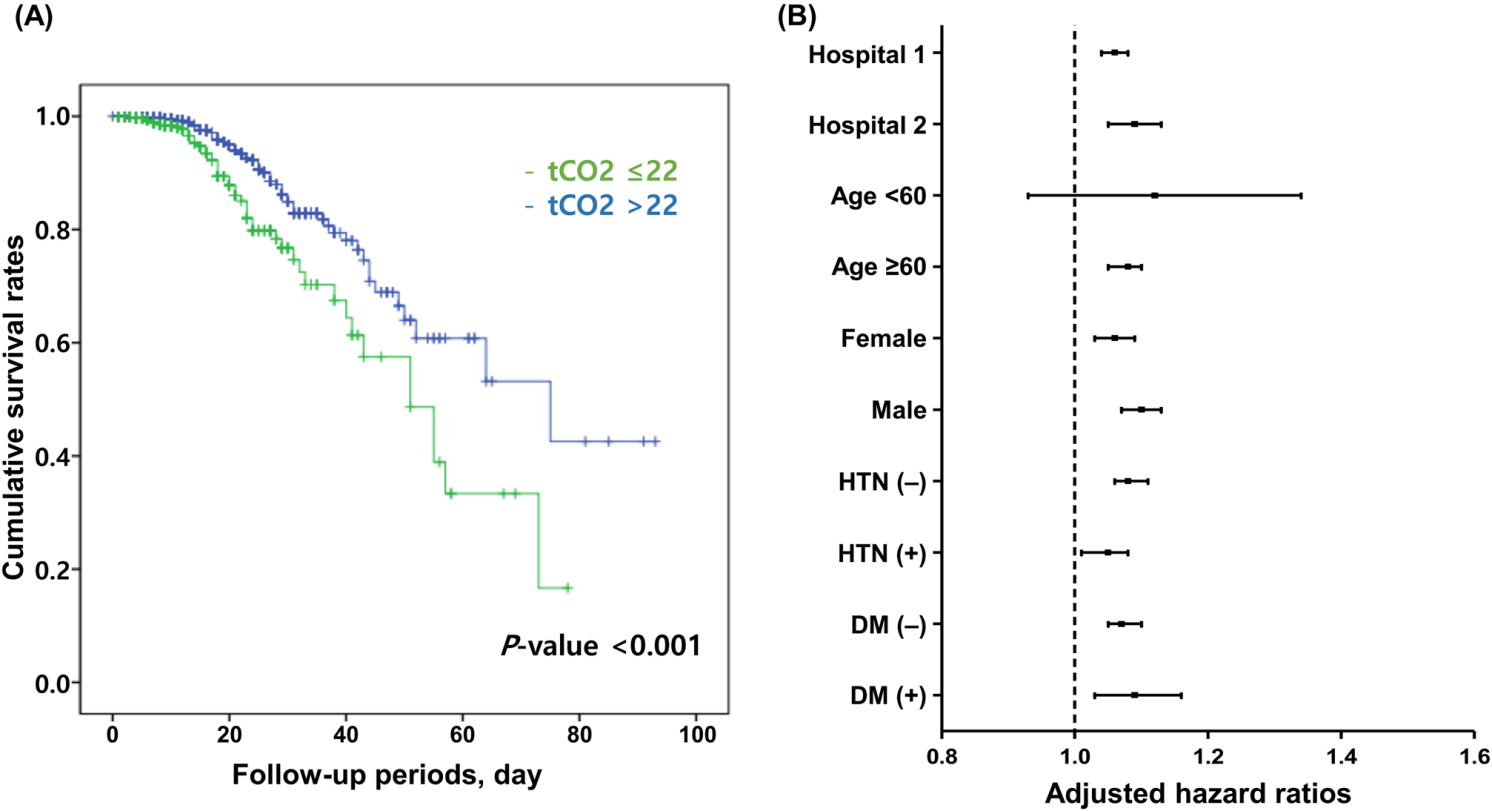
(**A**) Cumulative survival rate according to the 22 mmol/L cutoff of tCO_2_ and (**B**) subgroup analysis according to the each hospital, age 60 years, sex, and comorbidities such as hypertension and diabetes. Adjusted hazard ratios are shown with 95% confidence intervals. Adjusted variables in subgroup analyses: age, sex, white blood cell count, hemoglobin, platelets, calcium, glucose, blood urea nitrogen, eGFR, protein, albumin, uric acid, total cholesterol, total bilirubin, aspartate aminotransferase, C-reactive protein, hypertension, and diabetes.

**Figure 3:**
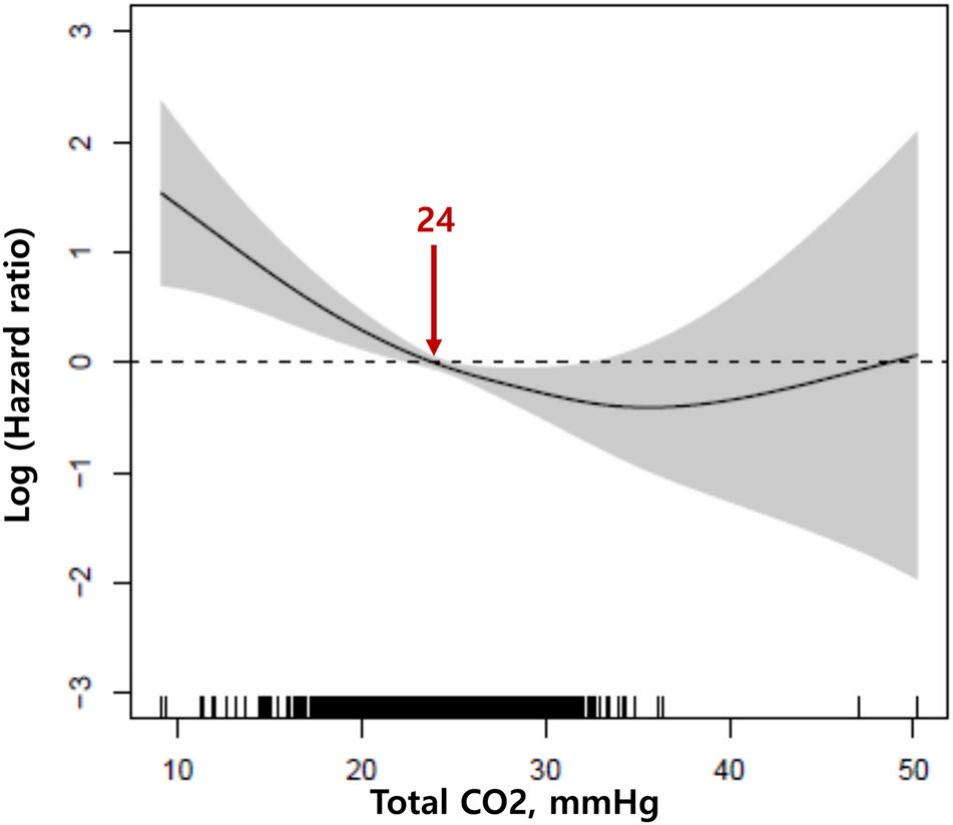
Cutoff value representing the increased risk of mortality based on the generalized additive model.

Based on the level of 22 mmol/L, the mortality rate was significantly different between group classified according to tCO_2_ level (Fig. 2A). This result was maintained irrespective of the each hospital (Fig. S1), sex, and comorbidities, such as hypertension and diabetes, in the subanalysis (Fig. 2B). In subjects with tCO_2_ ≤ 22 mmol/L, the mortality rate was significantly increased by 1.78 times compared to that in patients with tCO_2_ > 22 mmol/L. We additionally identified 24 mmol/L tCO_2_ as a new cutoff value indicating an increased mortality risk using a generalized additive model in this study (Fig. 3). Based on this new cutoff value, the mortality risk was well discriminated (Fig. S2). The risk of mortality (1.73 times) in subjects with tCO_2_ ≤ 24 mmol/L was similar to that in individuals classified according to the prior cutoff value of tCO_2_, 22 mmol/L (Table 3).

**Table 3:** Significance of tCO_2_ in terms of all-cause mortality based on the different cut-off value.

	Model 1		Model 2		Model 3		Model 4	
	HR (95% CI)	*P*-value	aHR (95% CI)	*P*-value	aHR (95% CI)	*P* value	HR (95% CI)	*P*-value
tCO_2_ ≤ 24	1.95 (1.37, 2.76)	< 0.001	1.65 (1.19, 2.30)	0.003	1.69 (1.21, 2.37)	0.002	1.73 (1.16, 2.58)	0.008
tCO_2_ ≤ 22	2.17 (1.53, 3.07)	< 0.001	1.76 (1.25, 2.47)	0.001	1.82 (1.28, 2.59)	< 0.001	1.78 (1.18, 2.67)	0.006

Model 1: Unadjusted.

Model 2: Adjusted for age and sex.

Model 3: Adjusted for variables in model 2 with comorbidities including hypertension, diabetes, cerebrovascular disease, cardiovascular disease, pulmonary disease, and acute kidney injury requiring kidney replacement therapy.

Model 4: Adjusted for variables in model 3 with laboratory variables including white blood cell count, hemoglobin, platelet, potassium, calcium, glucose, blood urea nitrogen, eGFR, protein, albumin, uric acid, total cholesterol, total bilirubin, C-reactive protein.

HR, hazard ratio; CI, confidence interval; aHR, adjusted hazard ratio; tCO2, total carbon dioxide.

### Relative factors associated with lower tCO_2_

Several factors were associated with tCO_2_ ≤ 22 mmol/L in patients with COVID-19 infection. Most variables were associated with lower tCO_2_ in the unadjusted model. However, after adjustment with significant variables in model 2, there was no association with age or sex on the lower tCO_2_. Among the laboratory parameters, higher WBC, lower hemoglobin, lower calcium, lower estimated glomerular filtration rate (eGFR), and higher uric acid, and AST were significantly associated with tCO_2_ ≤ 22 mmol/L in adjusted model. In particular, the significance of comorbidities including hypertension, diabetes, cerebrovascular disease, cardiovascular disease, and acute kidney injury requiring KRT was attenuated in the adjusted model (Table 4).

**Table 4:** Relative factors associated with tCO_2_ ≤ 22.

	Model 1		Model 2	
	OR (95% CI)	*P* value	aOR (95% CI)	*P* value
Age, years	1.02 (1.01, 1.02)	< 0.001	0.99 (0.99, 1.00)	0.086
Sex, male, n(%)	1.33 (1.14, 1.56)	< 0.001	1.04 (0.83, 1.30)	0.730
WBC, × 1,000/uL	1.11 (1.08, 1.14)	< 0.001	1.07 (1.03, 1.11)	< 0.001
Hemoglobin, mg/dL	0.88 (0.84, 0.91)	< 0.001	0.92 (0.86, 0.98)	0.014
Platelet, × 1,000/uL	0.998 (0.997, 0.999)	< 0.001	1.00 (1.00, 1.00)	0.212
Calcium, mg/dL	0.73 (0.67, 0.80)	< 0.001	0.70 (0.60, 0.80)	< 0.001
Phosphorus, mg/dL	1.00 (0.89, 1.12)	0.970		
Potassium, mmol/L	1.21 (1.03, 1.42)	0.019	1.05 (0.87, 1.28)	0.602
Glucose, mg/dL	1.004 (1.003, 1.005)	< 0.001	1.00 (1.00, 1.00)	0.904
BUN, mg/dL	1.04 (1.04, 1.05)	< 0.001	1.01 (1.00, 1.02)	0.054
eGFR, mL/min/1.73 m2	0.98 (0.98, 0.98)	< 0.001	0.99 (0.98, 0.99)	< 0.001
Protein, g/dL	0.68 (0.60, 0.77)	< 0.001	1.05 (0.83, 1.33)	0.672
Albumin, g/dL	0.49 (0.42, 0.57)	< 0.001	0.97 (0.70, 1.33)	0.831
Uric acid, mg/dL	1.37 (0.32, 1.42)	< 0.001	1.36 (1.30, 1.42)	< 0.001
Total cholesterol, mg/dL	0.99 (0.99, 1.00)	< 0.001	1.00 (1.00, 1.00)	0.657
Total bilirubin, mg/dL	1.28 (1.05, 1.56)	0.014	1.10 (0.84, 1.43)	0.491
AST, mg/dL	1.01 (1.00, 1.01)	< 0.001	1.01 (1.00, 1.01)	< 0.001
ALT, mg/dL	1.00 (1.00, 1.00)	0.103		
CRP, mg/dL	1.02 (1.01, 1.04)	0.005	1.01 (0.99, 1.03)	0.453
History of hypertension	1.28 (1.02, 1.61)	0.033	0.83 (0.60, 1.14)	0.250
History of diabetes	1.80 (1.39, 2.33)	< 0.001	1.30 (0.90, 1.85)	0.162
Cerebrovascular disease	1.92 (1.12, 3.29)	0.018	1.13 (0.56, 2.27)	0.738
Cardiovascular disease	1.93 (1.05, 3.54)	0.034	1.02 (0.49, 2.12)	0.964
Obstructive pulmonary disease	0.87 (0.45, 1.66)	0.668		
Acute kidney injury requiring KRT	5.21 (2.00, 13.53)	< 0.001	0.53 (0.15, 1.88)	0.328

Model 1: Unadjusted.

Model 2: Adjusted with variable with P < 0.05 in Model 1.

WBC, white blood cell; BUN, blood urea nitrogen; AST, aspartate aminotransferase; ALT, alanine aminotransferase; CRP, C-reactive protein; KRT, kidney replacement therapy; OR, odds ratio; aOR, adjusted odds ratip; CI, confidence interval.

### The overall distribution of acid–base status

A total of 350 patients underwent arterial blood gas analysis on their date of admission. Mean partial pressure of CO_2_ (PaCO_2_) and tCO_2_ were 35.2 ± 6.0 mmHg and 25.3 ± 3.9 mmHg, respectively (Table S3). According to the tCO_2_ 22 mmol/L, there was an insignificant difference in pH and partial pressure of oxygen (PaO_2_), but PaCO_2_, tCO_2_, and bicarbonate (HCO_3_) were significantly lower in patients with tCO_2_ ≤ 22 mmol/L. More than half of the patients (54.8%) showed alkalemia with pH ≥ 7.45. However, there were only 4 (1.1%) and 11 (3.2%) patients with pH ≥ 7.60 and pH < 7.35, respectively. Patients with alkalemia showed higher PaO_2_, lower PaCO_2_, and higher HCO_3_ than patients with normal pH (Table S4). The proportion of patients with tCO_2_ ≤ 22 mmol/L was lowest in patients with alkalemia. The correlation between changes in PaCO_2_ and HCO_3_ showed the proper compensation response to acid–base imbalance (Fig. S3). Among the 49 subjects with normal pH with HCO_3_ ≤ 24 mmol/L, the mean values of decreased tCO_2_ and PaCO_2_ were 3.8 mmol/L and 7.5 mmHg, respectively.

## Discussion

In the present study, a decreased level of tCO_2_, which is easily detected using serum samples, was found to be a significant risk factor for mortality. The mortality risk increased incrementally from the traditional cutoff level of tCO_2_ ≤ 22 mmol/L. Additionally, the level of tCO_2_ ≤ 24 mmol/L, which was confirmed by a generalized additive model, showed similar results. We found that increased inflammatory markers, and dysfunction in major organs, such as the kidneys and liver, were associated with decreased tCO_2_.

Metabolic acidosis is a risk factor for mortality, especially in critically ill patients^1,15^. Because of the variety of baseline conditions, it was challenging to identify the relationship between the type of acidosis and outcomes. In this regard, serum bicarbonate is commonly used as a variable representing acidosis status and mortality risk^16^. Considering the more convenient approach and excellent correlation with serum bicarbonate, tCO_2_ is widely used for monitoring acidosis status^8^. However, it needs to be a caveat concerning the interpretation of an isolated measurement. We suggest that the low levels of tCO_2_ were as a result of either metabolic acidosis or compensation for respiratory alkalosis.

Acid–base disorders are common in patients with COVID-19^17^, and the most frequently reported form is respiratory and metabolic alkalosis, especially in patients with a severe form of COVID-19^17,18^. Respiratory and metabolic alkalosis could be related to the disease characteristics of the respiratory infectious disease. Nevertheless, a severe form of metabolic acidosis was also linked to worse outcomes in terms of the cytokine storm, which contributes to the mortality of COVID-19^19^. In addition, a higher level of lactate, one of the major sources of metabolic acidosis, was significantly related to increased mortality in patients with COVID-19^20^. However, following the trends of increased prevalence and decreased case-fatality rate worldwide, a specific approach would be warranted to identify the factors with the ability to predict worse outcomes in patients with COVID-19 showing mild to moderate severity. In this regard, the present study determined the impact of lower tCO_2_ on in-hospital mortality for patients with mild to moderate COVID-19 and a susceptible pH level.

Metabolic acidosis may be indicative of infectious status, significant organ damage, and worsened clinical characteristics^21–23^. Higher WBC counts, CRP levels, and extended hospital stays were associated with the severity of infectious disease. In addition, decreased calcium, elevated creatinine, decreased eGFR, and elevated AST and ALT may be associated with impaired kidney and liver function. In the same context, a higher prevalence of comorbidities such as hypertension, diabetes, cerebrovascular disease, and cardiovascular disease could be considered^23^. In this study, we also found that patients with lower tCO_2_ had worse clinical characteristics.

The kidneys have been generally reported as an organ inducing metabolic acidosis and an organ affected by metabolic acidosis. Metabolic acidosis significantly increases mortality risk, especially in patients with advanced CKD^2,24^. We previously reported that these hazardous effects of metabolic acidosis on graft and patient survival were maintained in kidney transplant recipients^25^. Although most patients had preserved kidney function in this study, those with lower tCO_2_ showed lower eGFR, and a lower level of eGFR was a factor associated with tCO_2_ ≤ 22 mmol/L. Lower hemoglobin and calcium were significant factors associated with lower tCO_2_. Though a definitive causal relationship between these variables and acidosis could not be determined, all of these variables are closely related to kidney function status; therefore, a comprehensive approach including kidney function status would be required to interpret the results.

In addition to the kidney and lungs, the liver is a critical acid–base regulation organ^26^. Based on its role in lactate metabolism, ketogenesis, albumin synthesis, and urea production^27^, severe liver damage could be linked to metabolic acidosis^28^. In this study, 3.4% and 3.4% of patients showed AST and ALT over 3 times the upper normal limit, respectively. We found a significant negative association between the level of AST and tCO_2_. There was no statistical significance, but bilirubin also showed a negative association with tCO_2_. Considering the severity of the status of the included patients, it would be warranted to monitor major organ dysfunction and the status of acid–base disorder.

Although the lockdown has ended, the COVID-19 epidemic has continued. Acid–base imbalance could be regulated and managed; thus, evaluating and monitoring the acid–base status as a significant factor associated with mortality is essential. Considering the straightforward approach of assessing acid–base balance based on the single measure of tCO_2_, this study has the strength of suggesting unique and helpful guidance for general populations with mild-to-moderate severity COVID-19. However, there are several limitations to this study. First, this was a retrospective observational study. We could not figure out the exact causal association between each variable and acidosis status. In addition, it needs to consider the recall bias during the process of recruiting the diagnosis information. Second, we could not differentiate the detailed status of acid–base balance, including the compensation status. It is not a routine process to measure arterial blood gas; therefore, we could not fully obtain the data of all the variables, such as pH, PaCO_2_, and PaO_2_. Third, although COVID-19 infection is a respiratory infection, we did not consider respiratory alkalosis or distress because of the small number of patients requiring oxygen supply or mechanical ventilation in this study. Fourth, we analyzed the significance of tCO_2_ on mortality in only patients with COVID-19 infection. Therefore, we suggest that these results only applied to patients with COVID-19, not to compare the patients without COVID-19.

Decreased tCO_2_, especially in patients with tCO_2_ ≤ 22 mmol/L significantly increases the risk of mortality in patients with COVID-19 infection. Considering the socioeconomic burden of COVID-19 infection, identifying factors that can predict outcomes is helpful in distinguishing patients based on risk stratification. Evaluating and monitoring tCO_2_ based on a convenient approach could be an excellent method to predict mortality, especially in patients with mild-to-moderate severity COVID-19 infection.

## Materials and methods

### Study populations

We included subjects aged ≥ 18 years from two hospitals dedicated to COVID-19 treatment during February 2020 and September 2021. We excluded subjects with underlying end-stage kidney disease requiring KRT. Additionally, subjects without data on total CO_2_ value were excluded. All subjects were followed-up from the date of admission related to COVID-19 to discharge from the hospital. The type of discharge included discharge to home, transfer, and death.

### Clinical parameters and data acquisition

We collected laboratory data from the admission date, including complete blood counts; serum calcium, phosphate, serum potassium, and glucose; blood urea nitrogen; creatinine; protein; albumin; total bilirubin; aspartate transferase; alanine transferase; uric acid; total cholesterol; CRP; and lactate. The eGFR was calculated using the Chronic Kidney Disease Epidemiology Collaboration equation. We collected acid–base parameters of tCO_2_ and bicarbonate in whole populations. Moreover, we obtained arterial blood gas analysis data including pH, PaO_2_, and PaCO_2_ from those who had results. We also obtained information on underlying comorbidities such as hypertension, diabetes, cerebrovascular disease, cardiovascular disease, and chronic obstructive pulmonary disease based on the medication history and responses of the patients. We used tCO_2_ as an exposure variable, and a level of 23 mmol/L was regarded as a lower limit of the normal range^29^. The primary outcome was all-cause mortality during hospitalization.

### Sensitivity analysis

We additionally evaluated acid–base status in subjects according to arterial blood gas analysis. We divided the patients into three groups depending on the level of pH: < 7.35, ≥ 7.35 and < 7.45, and ≥ 7.5. The distribution of PaO2, PaCO2, bicarbonate, and tCO_2_ were evaluated according to pH.

### Statistical analysis

Continuous variables are presented as the mean and standard deviation (SD). Categorical variables are shown as a number with a percentage. To compare the two groups according to the level of tCO_2_, we used Student's t tests and chi-square tests. We performed a Cox proportional hazard analysis to identify the impact of tCO_2_ on all-cause mortality. We used logistic regression analysis to determine the factors associated with decreased tCO2. In the multivariate analysis, we adjusted these variables, including age, sex, comorbidities, and laboratory findings, including WBC counts, hemoglobin, platelets, serum calcium, glucose, albumin, eGFR, and CRP. We used a generalized additive model to identify the association of tCO2 with increased risk of all-cause mortality. *P* values < 0.05 were defined as significant when they were two-sided. Statistical analyses were performed using SPSS (version 23.0; IBM Corp., Armonk, NY, USA).

### Ethical consideration

This study was conducted after approval by the institutional review board of Seoul National University Boramae Medical Center (IRB No 20–2021-88). The study was performed in accordance with the principles of the Declaration of Helsinki. All of the clinical information was extracted by retrospective review, and informed consent was waived by the institutional review board of Seoul National University Boramae Medical Center (IRB No 20–2021-88).

### Supplementary Information

Supplementary Information.
