# Supplementary material for: Impact of decreased levels of total CO2 on in-hospital mortality in patients with COVID-19
Source: Sci Rep. 2023 Oct 4;13:16717. doi: 10.1038/s41598-023-41988-4 (PMC10550989; doi:10.1038/s41598-023-41988-4)

**Table S1**. Distribution of all parameters of arterial blood gas analysis according to the pH

| Comorbidities | n | tCO2 | *P*-value |
| --- | --- | --- | --- |
| Hypertension (-) | 3,901 | 24.84 ± 2.94 | 0.125 |
| Hypertension (+) | 522 | 24.62 ± 3.72 |  |
| Diabetes (-) | 4,090 | 24.86 ± 3.00 | <0.001 |
| Diabetes (+) | 333 | 24.21 ± 3.43 |  |
| Cerebrovascular disease (-) | 4,358 | 24.83 ± 3.04 | 0.015 |
| Cerebrovascular disease (+) | 65 | 23.90 ± 3.08 |  |
| Cardiovascular disease (-) | 4,372 | 24.83 ± 3.04 | 0.012 |
| Cardiovascular disease (+) | 51 | 23.75 ± 3.16 |  |
| Obstructive pulmonary disease (-) | 4,354 | 24.81 ± 3.04 | 0.273 |
| Obstructive pulmonary disease (+) | 69 | 25.2 ± 2.99 |  |

tCO2, total carbon dioxide

**Table S2**. Baseline characteristics according to survival status

| **Variables** | **Total (n=4,423)** | **Alive**  **(n=4278)** | **Death**  **(n=145)** | ***P*-value** |
| --- | --- | --- | --- | --- |
| Age, year | 54.7 ± 18.3 | 53.8 ± 17.8 | 81.4 ± 10.5 | <0.001 |
| Sex, male, n(%) | 1,935 (45.7) | 1,957 (45.7) | 70 (48.3) | 0.554 |
| WBC, x1,000/uL | 5.3 ± 2.5 | 5.2 ± 2.3 | 7.8 ± 5.4 | <0.001 |
| Hemoglobin, mg/dL | 13.4 ± 1.8 | 13.4 ± 1.8 | 12.1 ± 2.1 | <0.001 |
| Platelet, x1,000/uL | 193.9 ± 73.5 | 195.0 ± 73.0 | 162.4 ± 80.2 | <0.001 |
| Potassium, mmol/L | 4.0 ± 0.5 | 4.0 ± 0.5 | 4.2 ± 0.8 | <0.001 |
| Calcium, mg/dL | 8.7 ± 0.9 | 8.7 ± 0.8 | 8.2 ± 1.2 | <0.001 |
| Phosphorus, mg/dL | 3.2 ± 0.7 | 3.2 ± 0.7 | 3.4 ± 1.3 | 0.102 |
| Glucose, mg/dL | 129.2 ± 52.6 | 128.7 ± 51.7 | 146.1 ± 71.3 | 0.004 |
| BUN, mg/dL | 14.5 ± 10.7 | 139.9 ± 9.2 | 32.0 ± 25.8 | <0.001 |
| Creatinine, mg/dL | 1.0 ± 1.8 | 0.9 ± 1.1 | 2.3 ± 7.5 | 0.027 |
| eGFR, mL/min/1.73 m2 | 94.2 ± 25.0 | 95.3 ± 23.9 | 60.0 ± 30.5 | <0.001 |
| Total bilirubin, mg/dL | 0.5 ± 0.4 | 0.5 ± 0.4 | 0.7 ± 0.5 | <0.001 |
| AST, mg/dL | 37.0 ± 33.8 | 36.2 ± 29.7 | 58.0 ± 92.1 | 0.005 |
| ALT, mg/dL | 30.7 ± 33.4 | 30.6 ± 32.8 | 32.2 ± 47.5 | 0.571 |
| Protein, g/dL | 6.8 ± 0.6 | 6.8 ± 0.6 | 6.4 ± 0.8 | <0.001 |
| Albumin, g/dL | 4.0 ± 0.5 | 4.1 ± 0.5 | 3.5 ± 0.6 | <0.001 |
| Uric acid, mg/dL | 2.5 ± 2.5 | 2.5 ± 2.5 | 2.1 ± 3.3 | 0.108 |
| Total cholesterol, mg/dL | 152.1 ± 38.2 | 152.9 ± 37.5 | 131 ± 48.6 | <0..001 |
| C-reactive protein, mg/dL | 2.9 ± 4.4 | 2.8 ± 4.3 | 5.5 ± 5.3 | <0.001 |
| tCO2, mmHg | 24.8 ± 3.0 | 24.9 ± 3.0 | 23.4 ± 4.8 | <0.001 |
| History of hypertension, n(%) | 522 (11.8) | 475 (11.1) | 47 (32.4) | <0.001 |
| History of diabetes, n(%) | 333 (7.5) | 310 (7.2) | 23 (15.9) | 0.001 |
| Cerebrovascular disease, n (%) | 65 (1.5) | 56 (1.3) | 9 (6.2) | <0.001 |
| Cardiovascular disease, n (%) | 51 (1.2) | 38 (0.9) | 13 (9.0) | <0.001 |
| Obstructive pulmonary disease, n (%) | 69 (1.6) | 66 (1.5) | 3 (2.1) | 0.615 |
| Acute kidney injury requiring KRT | 17 (0.4) | 12 (0.3) | 5 (3.4) | <0.001 |
| In-hospital staying periods, days | 11.8 ± 8.5 | 11.5 ± 8.0 | 21.9 ±14.6 | <0.001 |

WBC, white blood cell; BUN, blood urea nitrogen; eGFR, estimated glomerular filtration rate; AST, aspartate aminotransferase; ALT, alanine aminotransferase; tCO2, total carbon dioxide; KRT, kidney replacement therapy

**Table S3**. Distribution of all parameters of arterial blood gas analysis according to the tCO2

| Variables | **Total (n=350)** | **tCO2>22 (n=303)** | **tCO2≤22 (n=47)** | *P*-value |
| --- | --- | --- | --- | --- |
| pH | 7.0 ± 0.8 | 7.0 ± 0.8 | 7.1 ± 0.7 | 0.365 |
| PaO_2_, mmHg, mean ± SD | 94.1 ± 9.5 | 93.9 ± 9.9 | 95.5 ± 6.1 | 0.298 |
| PaCO_2_, mmHg, mean ± SD | 35.2 ± 6.0 | 36.1 ± 5.5 | 29.3 ± 5.5 | <0.001 |
| tCO_2_, mmHg, mean ± SD | 25.3 ± 3.9 | 26.3 ± 2.9 | 19.0 ± 3.1 | <0.001 |
| HCO_3_, mmHg, mean ± SD | 24.1 ± 4.1 | 25.0 ± 3.3 | 18.4 ± 4.0 | <0.001 |

PaO2, partial pressure of dioxygen; PaCO2, partial pressure of carbon dioxide; tCO2, total carbon dioxide

**Table S4**. Distribution of all parameters of arterial blood gas analysis according to the pH

| Variables | <7.35 (n=11) | ≥7.35, <7.45 (n=147) | ≥7.45 (n=192) | *P*-value |
| --- | --- | --- | --- | --- |
| PaO_2_, mmHg, mean ± SD | 91.8 ± 11.9 | 92.6 ± 12.5 | 95.5 ± 5.8 | 0.010 |
| PaCO_2_, mmHg, mean ± SD | 32.4 ± 9.5 | 37.8 ± 6.3 | 33.3 ± 4.6 | <0.001 |
| tCO_2_, mmHg, mean ± SD | 19.9 ± 6.6 | 25.2 ± 4.1 | 25.8 ± 3.2 | 0.147 |
| tCO_2_ ≤22, n(%) | 5 (45.5) | 28 (19.0) | 14 (7.3) | <0.001 |
| HCO_3_, mmHg, mean ± SD | 14.9 ± 5.9 | 23.9 ± 4.2 | 24.8 ± 3.1 | 0.044 |

*P*-value means comparing subjects with pH ≥7.35, <7.45, and pH ≥7.45

**Figure S1**. Cumulative survival rate according to the level of 22 mmol/L of tCO2 in (A) and (B) hospitals


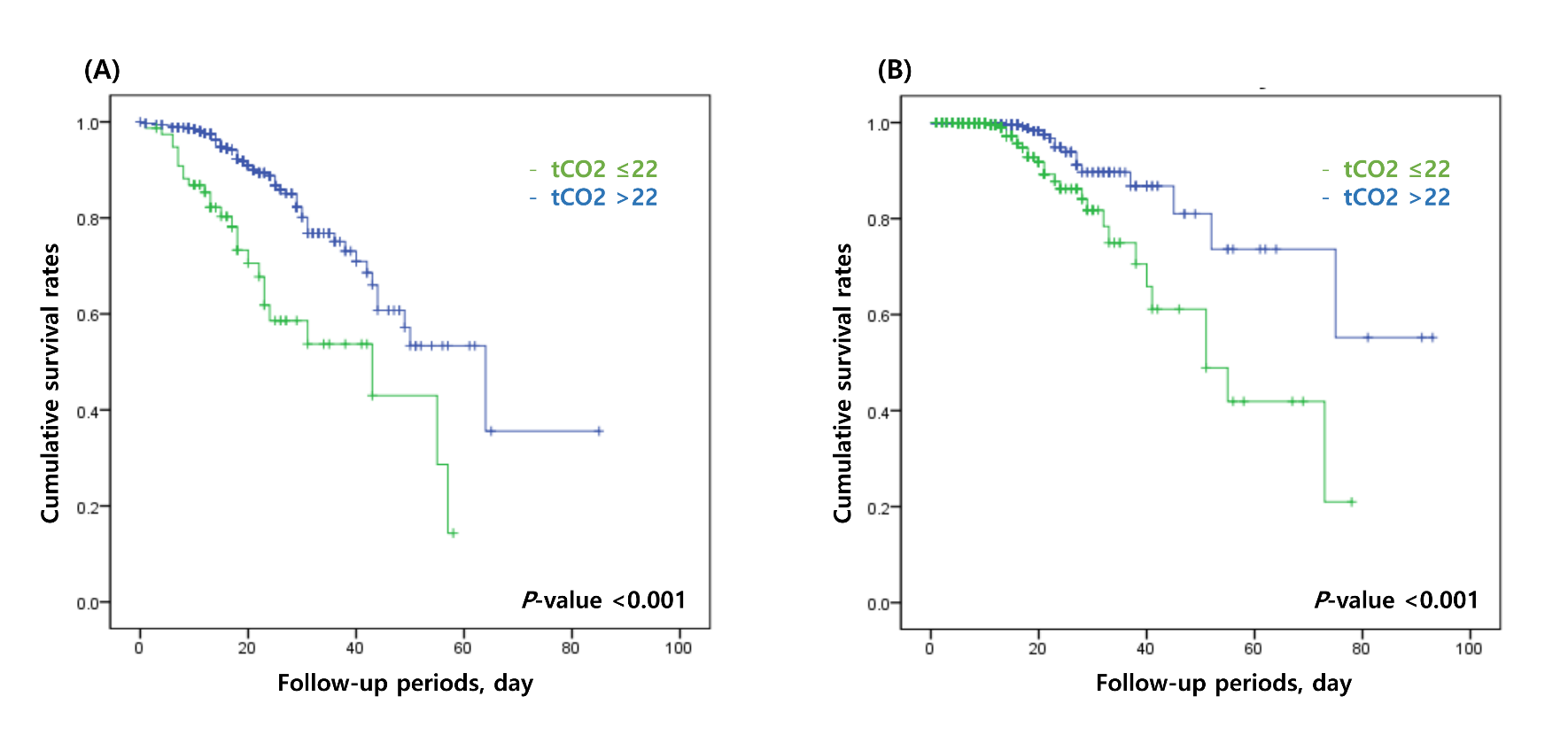


**Figure S2**. Cumulative survival rate according to the level of 24 mmol/L of tCO2


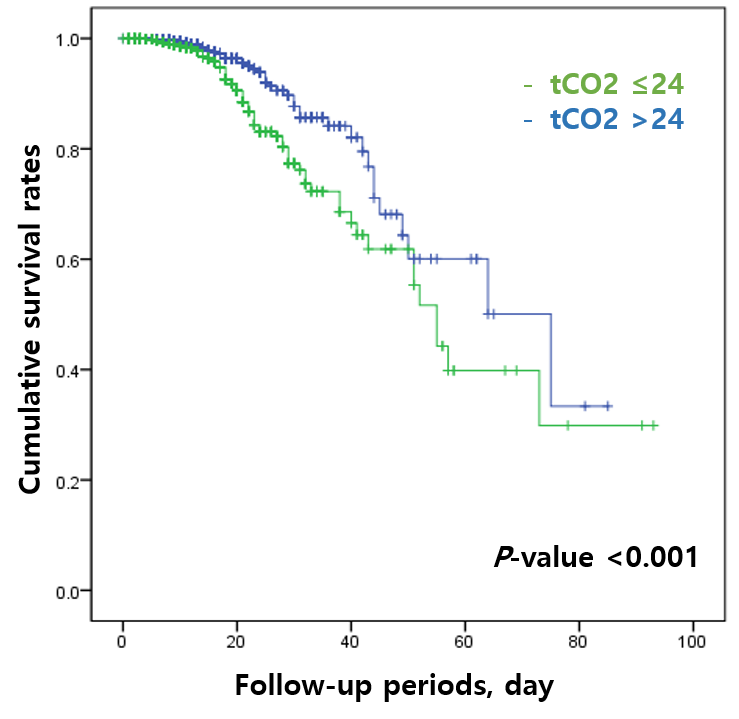


**Figure S3**. Correlation between changing in PaCO2 and HCO3 in patients (A) with and (B) without the alkalemia


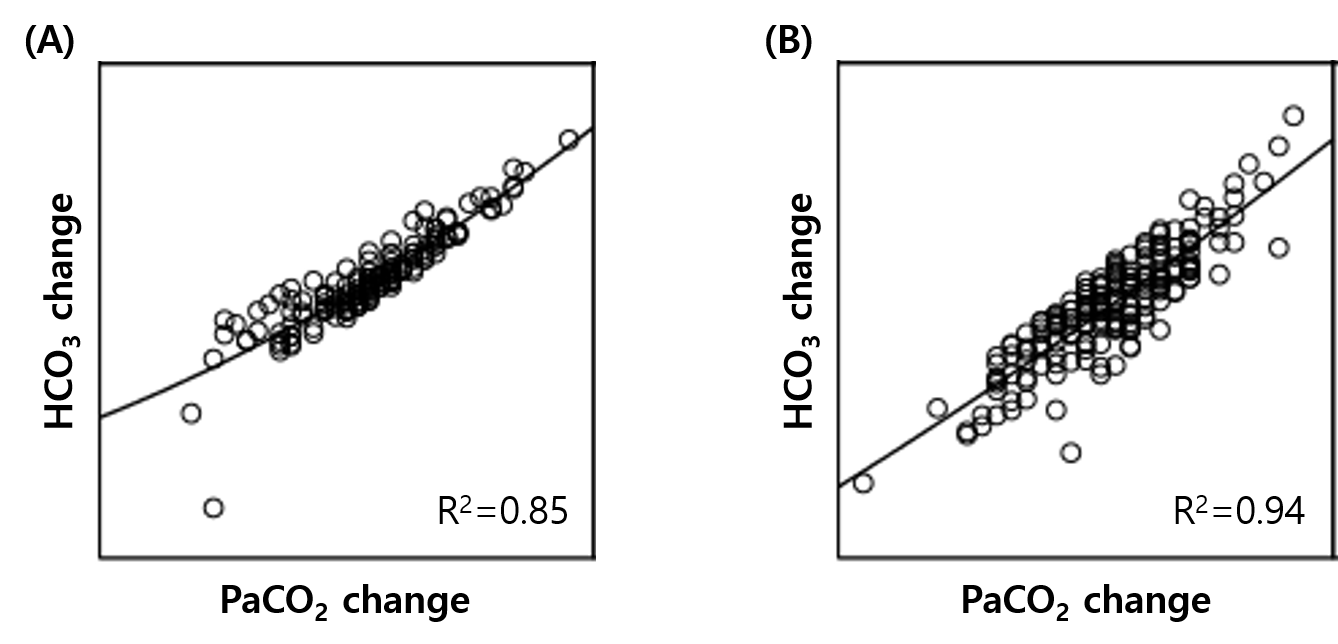

Supplement: Supplementary file 1 — Supplementary Information. [file 41598_2023_41988_MOESM1_ESM.docx]
